# Peer Review of “Social Media Polarization and Echo Chambers in the Context of COVID-19: Case Study”

**DOI:** 10.2196/32267

**Published:** 2021-08-05

**Authors:** Wayne Buente

**Affiliations:** 1 University of Hawaii at Manoa Honolulu, HI United States

**Keywords:** social media, opinion, COVID-19, case study, polarization, communication, Twitter, echo chamber

*This is a peer-review report submitted for the paper “Social Media Polarization and Echo Chambers in the Context of COVID-19: Case Study.”*

## Round 1 Review

### General Comments

This paper [1] addresses the issue of political polarization on social media during the COVID-19 pandemic. The study analyzes Twitter data, applying word content and social network analysis. The paper demonstrates the partisan polarity of users and influencers and the presence of echo chambers.

The paper focuses on the political polarization of Twitter users and makes an effective case for their presence and activities. However, the paper could provide a stronger connection to COVID-19 and public health implications. My thoughts are to have a section on COVID-19 and Twitter in the literature review. There have been infodemiology studies that might be useful to reference. It would be helpful to better situate the issue of political polarization of social media users and how it contributes to COVID-19. Why does it matter that political polarization and echo chambers exist for COVID-19 public health concerns? Similarly, there is no real connection to COVID-19 and public health implications in the Discussion section. How can the impressive findings of partisan Twitter users and echo chambers relate to COVID-19 health implications? I would like to see some connections made here to what we know about COVID-19 health and Twitter users.

Another concern is the highly technical methods of the study for Twitter data collection and analysis. I am familiar with Twitter scraping methods/analysis and social network analysis. However, the methodological techniques discussed are new to me. I would like to see better clarification on how these methods work.

### Specific Comments

#### Major Comments

1. The research questions (RQs) are fine for the study. There should be some connection between these 2 RQs and how they represent a “case study of COVID-19.”
2. On page 2, under “Related Work,” I would like to see an explanation of word embedding, network embedding, and transformers. I realize these are representation learning techniques to improve topic classification. It would be very helpful to have a basic explanation of what these techniques are doing that would be suitable for someone not in the computer science field. Even providing real-world examples would be helpful here. Since embedding and transformers are key parts of the methodology section, these techniques could use better explanation.
3. In the Methods section, I understand utilizing content analysis of profile words and retweet interactions to classify polarization of Twitter users in the data set. However, the specific techniques of average word embedding and transformers were hard to follow. I think it would be helpful to have a more layman’s definition of sentence embedding, transformers, and how they work in this data set. Perhaps a sample walkthrough of how a set of Twitter users is classified would be really beneficial in my opinion.
4. Under section 5.1, there is an analysis of bot scores (Figure 2B). Yet previously it was mentioned that the top 10% of users with a bot score were removed. So, is it still helpful to do this analysis? Can we still state that the presence of bots is being controlled in the Twitter data set?
5. Under section 5.2, the following is stated: “Figure 3 reveals the proportion of users in each decile of polarity score that are influential. We show that, consistent with all of the influence measures above, partisan users are more likely to be found influential.” Looking at Figure 3, only A and E really demonstrate this statement. Figure 3B, C, and D seem much more proportional (mild U shape).
6. In section 5.1, the classifications discovered are very interesting. These visualizations on partisanship and information dissemination are really nicely done. This finding is certainly a strength of the study. I also appreciate the visualizations for the polarization of influencers in section 5.2. It is helpful to see how partisanship contributes to information and influence in this Twitter data set.
7. I particularly like the Figure 6 visualization since it is the most intuitive of the visualizations.
8. I would like to see the COVID-19 health implications of these findings on the political polarization of Twitter users in the discussion section.

#### Minor Comments

1. On the first page, there is a reference to “AUC” without definition. Please define the acronym here.
2. In the “Transformers” paragraph, there is a reference to “NLP” without definition. Please define the acronym here.
3. In Figure 3, the caption states, “(B) top 10% in the number of followers,” but the graph heading shows the top 5%. I suspect the Figure 3 caption is incorrect.
4. Random Walk Controversy is an interesting data technique. I have never encountered it before.

## Round 2 Review

### General Comments

I appreciate the authors’ explanations for the reviewer comments. On reading the revised paper and the author feedback, I understand that this paper cannot address the COVID-19 tweet content since it appears that it is addressed in another work. As a study on the aspects of information and polarization in social media during COVID-19, I find the work to be much improved and enjoyed being able to review it.

## References

[1] Jiang J, Ren X, Ferrara E (2021). Social Media Polarization and Echo Chambers in the Context of COVID-19: Case Study. JMIRx Med.

